# Development and Validation of a Reproducible and Label-Free Surface Plasmon Resonance Immunosensor for Enrofloxacin Detection in Animal-Derived Foods

**DOI:** 10.3390/s17091984

**Published:** 2017-08-30

**Authors:** Mingfei Pan, Shijie Li, Junping Wang, Wei Sheng, Shuo Wang

**Affiliations:** Key Laboratory of Food Nutrition and Safety, Ministry of Education of China, Tianjin University of Science and Technology, Tianjin 300457, China; panmf2012@tust.edu.cn (M.P.); lsj2015@mail.tust.edu.cn (S.L.); wangjp@tust.edu.cn (J.W.); shengwei@tust.edu.cn (W.S.)

**Keywords:** enrofloxacin, surface plasmon resonance, immunosensor, animal-derived food samples

## Abstract

This study describes the development of a reproducible and label-free surface plasmon resonance (SPR) immunosensor and its application in the detection of harmful enrofloxacin (ENRO) in animal-derived foods. The experimental parameters for the immunosensor construction and regeneration, including the pH value (4.5), concentration for coating ENRO-ovalbumin conjugate (ENRO-OVA) (100 μg·mL^−1^), concentration of anti-ENRO antibody (80 nM) and regeneration solution (0.1 mol·L^−1^ HCl) were evaluated in detail. With the optimized parameters, the proposed SPR immunosensor obtained a good linear response to ENRO with high sensitivity (IC_50_: 3.8 ng·mL^−1^) and low detection limit (IC_15_: 1.2 ng·mL^−1^). The proposed SPR immunosensor was further validated to have favorable performances for ENRO residue detection in typical animal-derived foods after a simple matrix pretreatment procedure, as well as acceptable accuracy (recovery: 84.3–96.6%), precision (relative standard deviation (n = 3): 1.8–4.6%), and sensitivity (IC_15_ ≤ 8.4 ng·mL^−1^). Each SPR chip for analysis can be reused at least 100 times with good stability and the analysis cycle containing the steps of sample uploading/chip regeneration/baseline recovery can be completed within 6 min (one cycle) and auto-operated by a predetermined program. These results demonstrated that the proposed SPR immunosensor provided an effective strategy for accurate, sensitive, and rapid detection for ENRO residue, which has great potential for routine analysis of large numbers of samples for measuring different types of compounds.

## 1. Introduction

Quinolones are a class of antibacterial agents widely used for the prevention and treatment of many kinds of infectious diseases in human and animals. Enrofloxacin (ENRO, C_19_H_22_FN_3_O_3_), a third generation fluoroquinolone, possesses good inhibitory effects not only on Gram-positive and negative bacteria, but also on mycoplasma. It was reported that this antibiotic can combine with bacterial DNA-gyrase A to inhibit the cleavage and ligation of DNA-gyrase, leading to the inhibition of the duplication and transformation of DNA [1,2]. Therefore, ENRO is widely used for the treatment and prevention of pathogenic microorganism infections in animal husbandry [3,4]. As a special antibiotic for veterinary use, ENRO was reported to have two elimination pathways after entering the animal’s body. The majority of the ENRO intake (approximately 79.2%) can be excreted in original form by the kidneys, while the remaining part will deethylate to give the metabolite ciprofloxacin (CIPRO), which continues to have antibacterial and bactericidal effects effect in the animal’s body. Therefore, ENRO can inhibit bacterial infections on a broad-spectrum, long-acting and low-cost way during the animal breeding process [5,6,7]. However, the heavy use or abuse of ENRO also leads to some negative effects such as the induction of bacterial resistance and drug accumulation in edible animal tissues [8,9], which has resulted in severe damage to human health. To standardize the use of ENRO and ensure its residual concentration in animal-derived foods at an acceptable level, many countries and organizations have established a maximum residue limit (MRL) for ENRO in various edible animal tissues (FDA: prohibition of ENRO use in stockbreeding and poultry; EU: 30 μg·kg^−1^ in liver, kidney and muscle tissues [10]; WHO: 40 μg·kg^−1^ [11]; China: 100 μg·kg^−1^ in eggs and milk) [12]. Researchers from all over the world have established a series of methods with different principles for the detection of ENRO residues in various animal-derived food products.

To date, instrumental analysis based on chromatographic separation techniques [13,14] and immunoassays [15,16] based on antigen (Ag)-antibody (Ab) binding are the major analytical methods for ENRO measurement in food samples. In our previous studies, we prepared a molecularly imprinted biomimetic material [17] and polyclonal Ab with specific ability to recognize ENRO and developed relative instrumental and immunoassays/products for ENRO residue detection in food products. Facing a large number of complex food samples that need to be tested, modern instrumental analysis usually requires a relatively complex sample pretreatment process that does not meet the requirements of rapid detection [18,19]. Traditional immunoassays, such as enzyme- and fluorescence-linked immunosorbent assay (ELISA), based on the specific reaction of Ag and Ab usually require skillful operators and have inevitable deficiencies in automated analysis and widespread use. Additional and complicated labeled probes (enzyme [20], Au nanoparticles [21,22], quantum dots [23,24], etc.) are usually employed in immunoassays for signal enhancement, but the process is relatively complicated and can produce "false positive" results [25,26]. Therefore, there is a pressing need for and great interest in developing new analytical techniques with a simple pretreatment process, high degree of automation and high sensitivity, and low cost for the reliable detection and analysis of ENRO residues in food products.

Surface plasmon resonance (SPR) [27,28] is a special optical physical phenomenon involving incident light spread on the surface of an optical medium (metal dielectric) with different refractive indexes, whereby metal free electron resonance results and absorbs the photon energy, thus weakening the energy of the reflected light [29]. In this system, the resulting incident angle (defined as the SPR angle) at the time of plasma resonance is related to the mass of the biomolecule bound to the surface of the metal medium. Thus, according to the dynamic variation in incidence angle, the amount of biomolecules combined onto the surface of the metal medium can be monitored effectively, giving a specific signal reflecting the interactions between the biomolecules, which is the basic principle of SPR analysis [30,31]. An SPR biosensor is a new type of biosensing technology that has made great progress in recent years. Without the need for labeling and strict biological purification and purification processes, SPR biosensors can measure the interactions among all types of biomolecules such as proteins [32], DNA [33] and small molecule compounds [34] in real time, in situ and dynamically. SPR analysis can also monitor the binding of Ag and Ab on the surface of the chip and combine the high selectivity and sensitivity of immunoassays to improve the automation and stability in detection processes [35]. The combination of the SPR technique and the immunoassay, namely, the SPR immunosensor, has produced unparalleled advantages in the analysis of complex samples, especially in the fields of food analysis [36], environmental monitoring [37], medical testing [38] and other uses.

**Figure 1:**
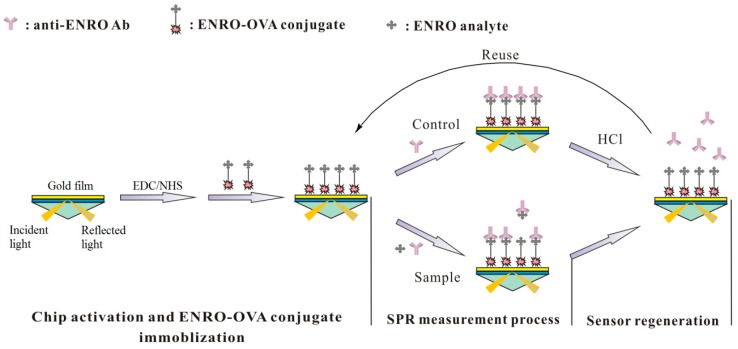
Schematic diagram of the SPR immunosensor for ENRO detection.

In this study, a reproducible, label-free SPR immunosensor was developed by a combination of SPR analysis and suppression immunoassay (Figure 1). It was demonstrated that the developed SPR immunosensing chip provided a reliable, stable, automated and low-cost analytical method for the detection of ENRO residue in animal-derived food products. This proposed SPR immunosensing detection mode can be further expanded to detect other targets in various fields.

## 2. Materials and Methods 

### 2.1. Reagents and Materials 

**Figure 2:**
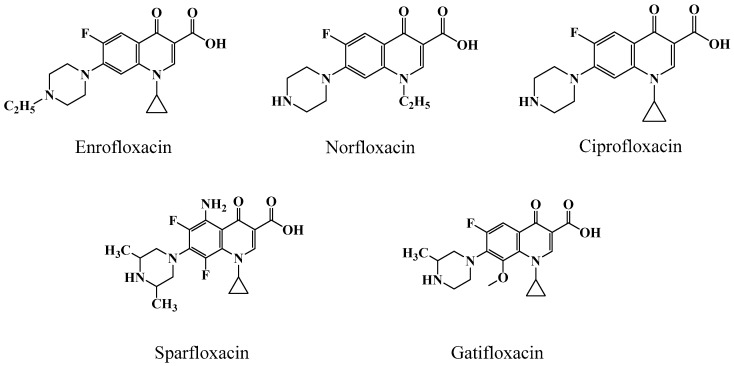
The chemical structure of target analyte ENRO and its analogues NOR, CIPRO, SPA and GAT.

The target analyte ENRO and its structural analogues used in the study (Figure 2), including norfloxacin (NOR), ciprofloxacin (CIPRO), sparfloxacin (SPA) and gatifloxacin (GAT) were purchased from Sigma-Aldrich (St. Louis, MO, USA) with guaranteed purity of more than 99.5 %. The compounds 1-(3-(dimethylamino)propyl)-3-ethylcarbodiimide hydrochloride (EDC), dicyclohexyl-carbodiimide (DCC) and N-hydroxysuccinimide (NHS) were also obtained from Sigma-Aldrich and used for the coupling of proteins on the surface of the sensor chip. The solutions of sodium dodecylsulfonate (SDS, 0.05%, w/v), surfactant P20 (5%, v/v), ethanolamine (1.0 mol·L^−1^, pH 8.5) and various buffers including HEPES buffered saline (HBS, 10×), glycine (Gly)-HCl (10 mM) and acetate buffer at different pH values (4.0, 4.5, 5.0 and 5.5) were purchased from General Electric Co., Ltd. (Tianjin, China).

Doubly deionized water (18.2 MΩ·cm) used in the experiment was prepared by a Millipore direct-Q ultrapure water system (Boston, MA, USA). NaOH, HCl, NaH_2_PO_4_ and other chemicals used in the experiment were obtained from Tianjin Chemical Reagent Factory (Tianjin, China). The selected animal food samples, such as pure milk, chicken muscle, beef, pork, and fish (grass carp), were all purchased from a local supermarket and determined to be free of ENRO before use by using HPLC-MS. 

### 2.2. Instrumentation

A biomacromolecular interaction analyzer (BIAcore 3000) from GE Healthcare Life Science (Tianjin, China) was employed for monitoring the binding reaction between the ENRO-OVA conjugate and anti-ENRO Ab on the SPR chip surface. The SPR sensor chip (CM500) wrapped with high-density carboxymethylated dextran matrix on the surface was purchased from GE Healthcare with two flow cells in a sequential array, of which one cell was used for the binding test and the other was treated as the control in the testing procedure. One vortex oscillation machine and a high-speed centrifuge (7200 g) from Sigma-Aldrich were used during the sample pretreatment procedure and the polypropylene millipore tubes (10.0 mL, cut-off molecular weight: 3000 Da) from Amicon Company (Bedford, MA, USA) were used to remove complex substances, such as proteins and other macromolecules.

### 2.3. Synthesis of ENRO-OVA Conjugate and Anti-ENRO Ab Preparation

#### 2.3.1. ENRO-OVA Conjugate

The mixed acid anhydride method [39] was used to synthesize ENRO-OVA using the following process. First, ENRO (21.5 mg) was accurately weighed and dissolved in anhydrous N,N-dimethyl-formamide (DMF, 3.0 mL) with continuous stirring in an ice-bath. Next, tri-*n*-butylamine (14.3 µL) and isobutyl chlorocarbonate (8.2 µL) were added to the stirring solution dropwise and then incubated for 1 h at 4 °C without light. Next, OVA solution (6 mL) containing OVA (50 mg) in 0.01 mol·L^−1^ carbonate buffer was added dropwise to the mixed solution. After incubation for 12 h at 4 °C without light, the mixture was dialyzed (molecular weight cut-off: 10,000 Da) against PBS (0.01 mol·L^−1^, pH 7.4) at 4 °C for 3 days. Finally, the product was centrifuged at 5000 r·min^−1^ at 4 °C, and the supernatant was determined as 3.15 mg·mL^−1^ using a BCA Protein Assay Kit and stored at 4 °C after adding 0.05% NaN_3_.

#### 2.3.2. Anti-ENRO Ab 

The activated ester method was used to prepare the immunogen ENRO-Keyhole Limpet Hemocyanin (KLH). Briefly, ENRO (8.9 mg), NHS (3.5 mg) and DCC (12.4 mg) were dissolved in DMF (150 μL) and shaken for 4–6 h in room temperature. Then, the solution was centrifuged for 5 min and the supernatant activated ester solution collected. The solution was added dropwise to NaHCO_3_ solution (3 mL, 130 mmol·L^−1^, pH 8.1) with KLH (10 mg), and reacted at 4 °C overnight. The ENRO-KLH was dialyzed for 3 days and stored at 4 °C. Next, soluble ENRO-KLH (1.0 mg) was mixed with Freund’s adjuvant (1:1, v/v; complete Freund’s adjuvant for first immunization and incomplete Freund’s adjuvant for booster immunization), emulsified completely and injected a healthy New Zealand white rabbit with the age of 3 months and the weight of 1.5 kg by subcutaneous injection every two weeks. After the two injections, a small quantity of blood was taken from marginal ear vein a week after injection, and tested by ELISA for the determination of antiserum titer and inhibition ratio. When the antiserum titer is appropriate, blood was collected by carotid puncture of the animal 5–7 days later after immunization. The collected blood was purified using protein A-Sepharose 4B affinity chromatography with a final concentration of 1.18 mg·mL^−1^ (approximately 80 μmol·L^−1^) and stored at 4 °C.

### 2.4. Immobilization of ENRO-OVA Conjugate on the SPR Chip Surface

The ENRO-OVA conjugate was immobilized onto the CM500 chip surface using the activating EDC and NHS agents. First, a mixed solution (100 μL) containing an equal volume of EDC (200 mmol·L^−1^) and NHS (50 mmol·L^−1^) was injected into the flow cell using two channels (one for the test and the other for the control) at a steady rate of 30 μL·min^−1^ (approximately 3 min) under room temperature. After rinsing with phosphate buffer solution (PBS) containing 0.5 % surfactant P20 for 2 min at 30 μL·min^−1^, the ENRO-OVA conjugate at a concentration of 100 μg·mL^−1^ in acetate buffer (pH 4.5) was continuously injected into the flow cell for immobilization onto the chip surface until the SPR response achieved to 1700 RU. Furthermore, the ethanolamine solution (1.0 mol·L^−1^, pH 8.5) was injected at a steady rate of 10 μL·min^−1^ for 10 min to block the unreacted sites on the sensor chip surface. Finally, the CM500 chip surface was washed using PBS containing 0.5% surfactant P20 until the SPR response was stable. Next, the SPR chips with ENRO-OVA conjugate after immobilization were stored at 4 °C before use. 

### 2.5. Measurement Procedure of ENRO Residue

In this study, the SPR immunosensor was designed based on the principle of immunosuppression for the detection of ENRO, and the whole detection procedure can be briefly described as follows: standard solutions of ENRO in PBS (pH 7.4) at different concentrations (0.05, 0.25, 0.5, 1.0, 2.5, 5.0, 7.5 and 10.0 ng·mL^−1^) or the sample extracts were first incubated with the anti-ENRO Ab (80 nM) for 5 min at room temperature. Next, the mixture of ENRO and anti-ENRO Ab was controllably injected into the flow cell at a steady rate of 30 μL·min^−1^ individually for 3 min to bind the unbound Abs with the ENRO-OVA conjugate immobilized on the CM500 chip surface. The resulting SPR response was recorded by the BIAcore data system and used for calculation of the inhibition ratio. After each SPR analysis, the HCl (0.1 mol·L^−1^) solution was pumped onto the chip surface at a 30 μL·min^−1^ rate for 1 min for regeneration and the PBS containing 0.5% P20 was further used to recover the stable baseline for next analysis (about 1 min). The analysis cycle of sample uploading/chip regeneration/baseline recovery was auto-operated by BIAcore 3000 Control Software. 

### 2.6. Samples Pretreatment and Spiked Method

In this study, typical animal-derived foods from a local supermarket including milk, chicken muscle, beef, pork and fish (grass carp) were analyzed by the developed SPR immunosensor after a simple pretreatment process. Accurately measured pure milk (5.0 mL) was placed into a polypropylene Millipore tube and centrifuged for 10 min at 4000 rpm at room temperature for removal of biological macromolecules such as proteins and lipids. The supernatant was separated and diluted 10-fold using deionized water and filtered through a 0.22 μm filter. After 10-fold dilution using PBS (pH 7.4) again, the liquid product was collected and marked as sample extract for further analysis. The solid food samples were pre-treated as follows: pre-crushed solid food samples (5.0 g) was accurately weighed into individual PTFE tubes. After adding the solvent acetonitrile (10.0 mL) to each tube, the mixture was shaken using a vortex machine for 10 min to be mixed fully for protein precipitation. After centrifugation using a polypropylene Millipore tube at 4000 rpm for 10 min, the supernatant was separated and diluted to a suitable volume using PBS (pH 7.4) for further research. 

In the matrix effect elimination experiments, various samples were spiked with ENRO of various concentrations and pretreated as above for SPR analysis to obtain the matrix standard curve of ENRO. In the spiked and recovery experiment, the chosen food samples were previously spiked at three levels (10.0, 25.0 and 50.0 ng·mL^−1^ or ng·g^−1^) and further pretreated as above and tested to validate the accuracy and precision of the developed SPR chip to analyze the ENRO residues.

## 3. Results and Discussion

### 3.1. Synthesis of ENRO-OVA Conjugate 

The ENRO-OVA conjugate used in this study was synthesized using the mixed acid anhydride method and UV scanning under full wavelength was employed to validate the results. As shown in the Electronic Supporting Materials (ESM 1), the analyte ENRO (blue) shows strong adsorptions at the wavelength of 270 nm and 330 nm, and the other reactant OVA (black) has apparent adsorption at 280 nm. The purified ENRO-OVA conjugate shows obvious adsorption at both 280 nm and 330 nm. This result demonstrated that the ENRO-OVA conjugate was successfully synthesized and could be used as a ligand for SPR chip modification. The concentration of ENRO-OVA conjugate was determined as 3.12 mg·mL^−1^. 

### 3.2. Immbolization of the ENRO-OVA Conjugate on the Sensor Chip

Detection using SPR is usually performed by modifying certain ligands on the surface of the chip for binding the target analyte, and the amount or concentration of the ligand largely depends on the coupling level on the SPR chip surface. A higher coupling level implies a larger binding response between the ligands and the analyte, achieving a higher sensitivity for detection. The surface of the SPR CM500 chip used in this study was wrapped by a carboxymethylated dextran matrix which contributed to the immobilization of the ligands. Compared to the SPR chip used before (CM5) [40], the selected CM500 chip has a higher density of binding cites, contributing to a larger amount of ligands immobilized on the surface. Simultaneously, this type of chip can provide a more stable response signal and a smaller non-specific adsorption, which is suitable for the construction of an SPR sensor with high sensitivity and stability.

For the microenvironment (pH value) of the binding reaction, it is usually necessary to bind the protein conjugate onto the surface of the chip as quickly as possible while simultaneously keeping as close as possible to a neutral environment to ensure the activity of the coupling protein. The carboxymethylated dextran matrix on the CM500 chip surface was first activated using EDC/NHS and applied to immobilize the ENRO-OVA conjugate. 

**Figure 3:**
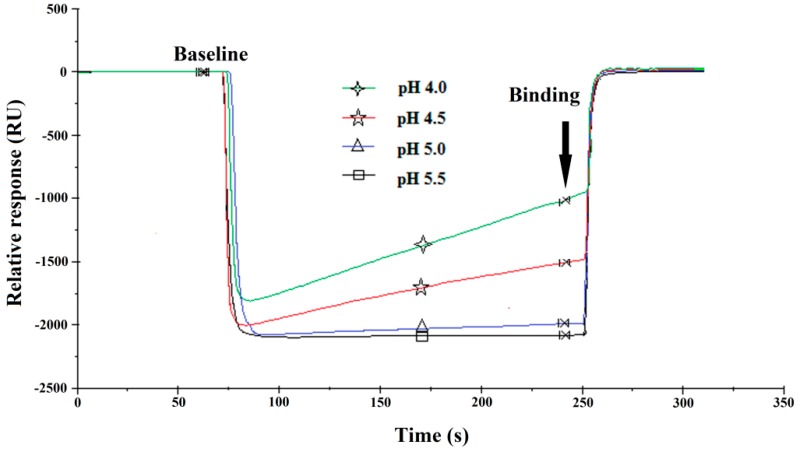
Results of the ENRO-OVA conjugate immobilized on the SPR chip in 10 mM acetate buffer solution at different pH values.

According to the pKa (3.5) of the carboxyl dextran and the pI (4.7) of the coupling protein (OVA), different pH values (4.0, 4.5, 5.0 and 5.5) of the acetate buffer solution (10 mM) were tested as the reaction microenvironment for ENRO-OVA immobilization on the chip surface (Figure 3). It was found that when the acetate buffer solution had a higher pH (5.0 and 5.5), almost no response was observed meaning that less ENRO-OVA conjugate was immobilized on the chip surface. This result can be explained by the fact that under the tested pH values of 5.0 and 5.5, the layer of carboxyl dextran and the coupling protein OVA both had a negative charge, resulting in electronic repulsion, which hindered the binding process on the chip surface. When the pH of the acetate buffer solution was controlled at 4.0 and 4.5 between the pKa and pI value, an obvious SPR response was observed due to the electrostatic adsorption between the negatively charged layer and the positively charged OVA protein. Furthermore, to ensure high activity of the coupling protein OVA, the acetate buffer solution at pH 4.5 was selected for ENRO-OVA conjugate immobilization on the chip surface. 

**Figure 4:**
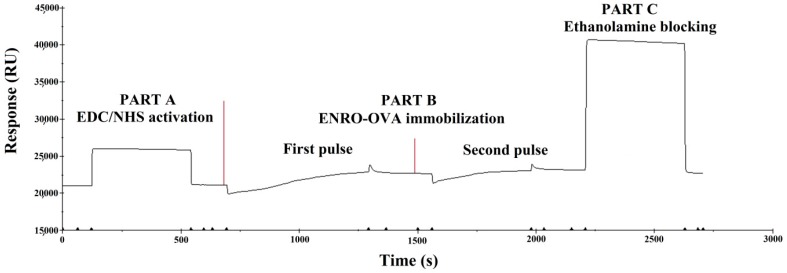
The immobilization of ENRO-OVA conjugate on the SPR chip surface. PART A: EDC/NHS activation process, PART B: ENRO-OVA immobilization (two binding pulses), PART C: Ethanolamine blocking process.

Binding of excess ligand (coupling protein) onto the surface of the chip will affect the accuracy of the SPR analysis. In the experiment, a predetermined binding response value on the SPR sensor was previously achieved through multiple injections of a certain concentration (100 μg·mL^−1^) of ENRO-OVA in acetate buffer (pH 4.5). After two binding pulses (Figure 4), the SPR response can be achieved at a predetermined value (1700 RU), and a suitable ENRO-OVA conjugate was immobilized on the chip surface. 

### 3.3. Optimization of Anti-ENRO Ab Concentration

**Figure 5:**
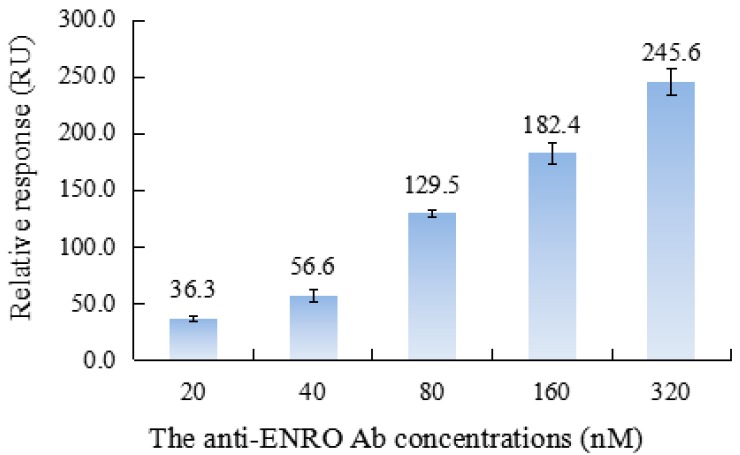
SPR response from the binding of the ligands with anti-ENRO Ab at different concentrations.

In the immunoassay, the concentration of the Ab largely affected the sensitivity and stability of detection and the chip regeneration and cost for detection, which was a crucial experimental parameter that needed to be evaluated. In the experiment, the anti-ENRO Ab solution at gradient diluted concentrations of 20, 40, 80, 160 and 320 nM were individually injected into the flow cell at a steady rate of 30 μL·min^−1^ for 3 min. The obtained SPR response was recorded by the Biacore 3000 Control Software. Figure 5 shows the comparison of the obtained SPR response. 

As shown, with the increase of the tested Ab concentration, the response on the SPR chip increased significantly, whereby the Ab concentration was 320 nM, and the SPR response can reach 245.6 RU, which can be explained by the increase of Ab concentration causing more Ab molecules to bind with the ligands on the chip surface. According to the resulting relative responses under different concentrations of anti-ENRO Ab, the binding constant K_D_ was calculated to be 1.73 × 10^−11^ M, signifying the good binding capacity of the ligands and the anti-ENRO Ab, which functions in the detection process of this proposed suppression SPR immunosensor. Additionally, too much Ab immobilized on the chip surface by the ligands is not only a waste of Ab, but can also lead to weak immune binding and furthermore the instability of the SPR signal response. The relative standard deviation (RSD) value for three paralleled bindings reached 9.5% (160 nM) and 11.2% (320 nM). As a result, the anti-ENRO Ab concentration was established as 80 nM for further research with a larger initial SPR response (129.5 RU) and good stability (RSD = 3.3%, n = 3).

### 3.4. Anti-ENRO Ab Dissociation for SPR Chip Regeneration

For the chips for be reused, a suitable regeneration procedure is a crucial step, which requires that the solution used for regeneration is not only be effective in removing the molecules bound onto the chip surface in the previous analysis, but it should also not disrupt the chip surface ligands to avoid affecting subsequent uses. In the experiment, different solutions for chip regeneration including HCl (0.1 mol·L^−1^), Gly-HCl (0.01 mol·L^−1^, pH 1.5), NaOH (0.05 mol·L^−1^), and SDS (0.5%) were compared to evaluate the stability and reusability of the SPR chips with the same concentration of anti-ENRO Ab (80 nM). 

**Figure 6:**
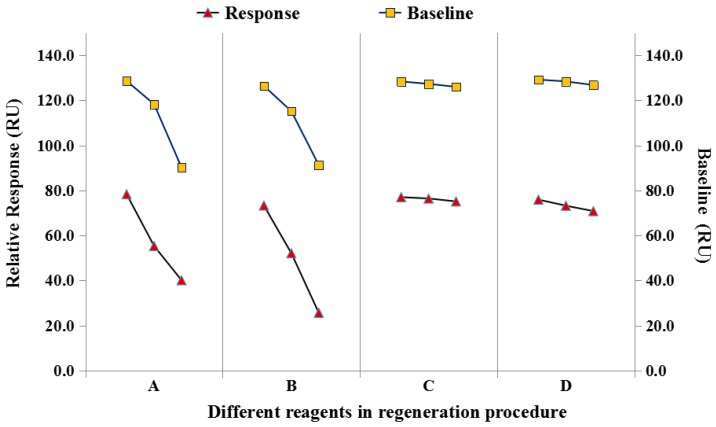
Comparison of baseline and relative response using different regeneration reagents: (A) 0.05 mol·L^−1^ NaOH; (B) 0.5% SDS; (C) 0.1 mol·L^−1^ HCl; (D) 0.01 mol·L^−1^ Gly-HCl (pH 1.5).

From the obtained results (Figure 6), it was observed that the SPR baseline (relative value) clearly decreased when the regeneration solution (A: 0.05 mol·L^−1^ NaOH; B: 0.5% SDS) was used, which can be explained by the damage of the ligand layer (ENRO-OVA) on the chip surface by the regeneration solution when the anti-ENRO Ab was removed in the regeneration procedure. When HCl (0.1 mol·L^−1^) (C) and Gly-HCl (0.01 mol·L^−1^, pH 1.5) (D) were used for chip regeneration, the baseline response on the SPR chip did not change significantly, and after three consecutive cycles, the SPR response for the tested Ab concentration (80 nM) was attenuated by 2.4% (C) and 6.7% (D), respectively. These results indicated that when HCl (0.1 mol·L^−1^) was used as the regeneration solution, it could effectively remove the binding protein and caused little damage to the ENRO-OVA conjugate. Thus, the HCl (0.1 mol·L^−1^) solution was selected for regeneration on the SPR chip surface. When the anti-ENRO Ab (80 nM) was applied for analysis, nearly 100 analysis cycles could be performed on one ENRO-OVA-immobilized SPR chip within one month, showing the high stability and reusability of the sensor surface. This result also signified that the developed detection chip has great potential for continuous, stable and low-cost analysis.

### 3.5. Measuring Procedure for ENRO and Cross-reactivity

According to the principle of immune suppression, standard working solutions with a series of ENRO concentrations (0.05, 0.25, 0.5, 1.0, 2.5, 5.0, 7.5 and 10.0 ng·mL^−1^) were individually mixed with anti-ENRO Ab (80 nM) for analysis using the developed SPR immunosensor. After mixing for inhibition for 5 min, the mixture of anti-ENRO Ab and ENRO was injected into the flow cell using a series system from Channel 2 to Channel 1, in which channel 1 was employed as a control to reduce the non-specific adsorption and remove the bulk effect from the different refractive indices of the instrumental buffer and sample solution. 

**Figure 7:**
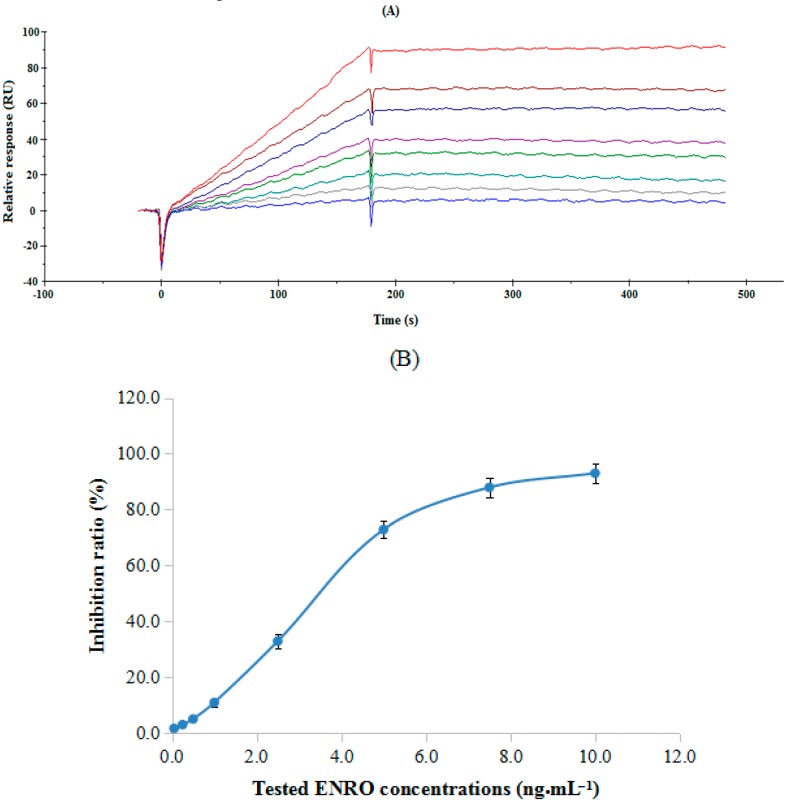
(**A**) SPR response from ENRO working solutions at different concentrations; (**B**) Inhibition curve of ENRO in PBS (pH 7.4) using the developed SPR immunosensor.

Figure 7 illustrates the SPR response (A) to the tested ENRO concentrations and the calculated inhibition curve (B). Under the optimized experimental conditions, the 50% inhibitory concentration (IC_50_) for sensitivity and 15% inhibitory concentration (IC_15_) for the detection limit for the ENRO analyte were 3.8 ng·mL^−1^ and 1.2 ng·mL^−1^, respectively. In the study, the specificity for ENRO detection using the developed SPR immunosensor was also evaluated using selected structurally related ENRO analogues at various concentration ranges. The percent cross-reactivity (%CR) was calculated as IC_50_ for ENRO/IC_50_ for the tested analogues. According to the obtained data (ESM. 2), the %CR of the selected structurally related ENRO analogues were calculated as 0.4% (NOR), 1.1% (CIPRO), 0.9% (SPA), 0.9% (GAT), respectively, which were all approximately 1%, indicating that the developed SPR immunosensor had higher specificity for ENRO detection.

### 3.6. Sample Matrix Effect and Recovery Studies 

Animal-derived foods are complex biological samples containing various biomolecules such as proteins and lipids, which could affect the accuracy and sensitivity of the detection of SPR immunosensors. In this study, the matrix effect from selected animal-derived food samples was evaluated in order to identify a relatively simple pretreatment process to eliminate the adverse effects from the matrix samples in the detection process. The chosen samples were spiked with ENRO at concentrations of 0.5 to 100 ng·mL^−1^ (or ng·g^−1^) and extracted during the matrix pretreatment process to prepare a series of ENRO working solution for SPR analysis, and the inhibition ratio was calculated according to the resulting SPR response. 

**Figure 8:**
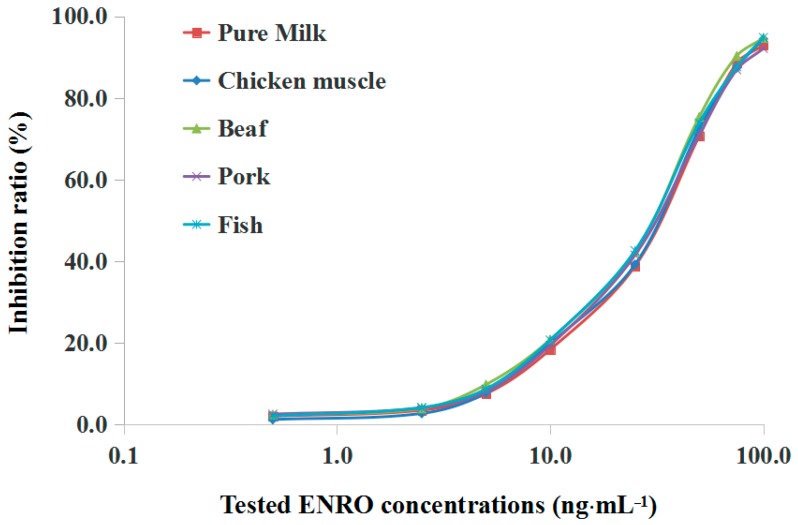
Calibration curves of chosen matrix samples using the developed SPR immunosensor.

As illustrated in Figure 8, it was observed that the inhibition ratios at each tested concentration in all selected samples were close to the corresponding values in the standard working solution (PBS), and the inhibition curve also had a similar trend at all tested concentrations. These results indicated that the matrix extract from chosen samples did not affect the binding of anti-ENRO Ab to the immobilized ENRO-OVA conjugate on the chip surface and demonstrated that this relatively simple pretreatment process for animal-derived food samples has been effective and promising in the application of a simple, rapid SPR analysis process. The IC_15_ and IC_50_ values for selected animal-derived foods achieved 8.4 and 34.2 ng·mL^−1^ (milk), 7.7 and 32.0 ng·g^−1^ (chicken muscle), 7.2 and 34.0 ng·g^−1^ (beef), 8.0 and 32.3 ng·g^−1^ (pork) and 7.5 and 30.1 ng·g^−1^ (fish), respectively, signifying that the developed SPR immunosensor provided a sensitive immunoassay for ENRO residue in real samples.

To further validate the accuracy and precision of SPR continuous analysis in real samples, the selected food samples were spiked at three levels (10.0, 25.0 and 50.0 ng·mL^−1^ or ng·g^−1^) and analyzed using the developed SPR immunosensor. The recoveries of ENRO and RSDs of analysis were calculated according to the SPR response in the range of 84.3 to 96.6% and 1.8 to 4.6% (Table 1). Furthermore, by comparison of the obtained results, it was found that the SPR response from continuous and paralleled ENRO determination at one concentration in various food samples did not show significant deviation with SD values less than 5%, meaning that the previous SPR assay did not affect the later assays. The same SPR chip can be reused for at least 100 analysis cycles in a favorable stability. Each analysis cycle can finish within 6 min including sample uploading (3 min), chip regeneration (1 min) and baseline recovery (approximately 1 min). The merits of this proposed SPR immunoassay for ENRO detection were compared to those from other researchers, and the results are illustrated in Table 2. These results demonstrated that the developed SPR immunosensor was feasible for providing accurate, sensitive, automated, high-throughput quantitative and qualitative analyses of ENRO residue in animal-derived foods using a simple preparation method. 

## 4. Conclusions

In this study, a label-free and reproducible SPR immunosensor based on the inhibition format was successfully constructed. This new SPR immunosensor combined the merits of SPR and immunoassay techniques and provided an accurate, sensitive, stable, automated and high throughput strategy for the detection of ENRO residues in animal-derived food samples. The detection strategy used in this study can also be further extended to the analysis and testing of other targets in various samples, demonstrating its very promising future in the field of automatic and continuous qualitative and quantitative analysis.

## Figures and Tables

**Table 1:** Recoveries for ENRO in spiked food samples analyzed by the developed SPR immunosensor and the commercial ELISA kit.

Sample	Spiked Levels (ng·mL^−1^ or ng·g^−1^)	SPR Immunosensor		Commercial ELISA Kit	
		Recovery (%)	RSD (%, n = 5)	Recovery (%)	RSD (%, n = 3)
Pure milk	10.0	88.7	3.0	113.7	8.7
	25.0	92.4	2.5	90.1	6.1
	50.0	96.3	3.9	118.8	10.1
Chicken muscle	10.0	84.3	1.8	80.3	2.3
	25.0	91.2	2.8	88.8	4.1
	50.0	92.7	4.6	78.1	4.4
Beef	10.0	87.8	3.8	89.3	2.8
	25.0	93.3	4.5	104.6	5.2
	50.0	95.6	2.8	91.3	5.5
Pork	10.0	92.5	3.9	103.9	8.8
	25.0	95.3	3.6	96.8	5.9
	50.0	96.2	2.6	90.9	5.2
Fish	10.0	89.3	3.3	101.7	2.3
	25.0	94.6	3.9	88.7	5.1
	50.0	96.6	3.6	100.2	6.8

**Table 2:** Comparison of different methods for ENRO determination in various samples

Methods	Sensitivity	LOD	Required Time	Reuse Cycles	Samples	References
Direct ELISA	20.0 ng·g^−1^	1.3 ng·g^−1^	>4.5 h	Once	feed	[15]
Quantum dot-based fluoroimmunoassay	1–100 ng·mL^−1^	2.5 ng·mL^−1^	>30 min	Once	Chicken muscle	[23]
Surface-enhanced Raman spectroscopy	-	10 ng·mL^−1^	40 min	Once	Chicken muscle	[41]
Microarray analyses	-	5 ng·kg^−1^	>1 h	-	Beef, pork and chicken	[42]
Immunoassay strip	0.038–22.75 ng·mL^−1^	0.935 ng·mL^−1^ (by scanner and eye)	5 min	Once	Chicken muscle	[43]
Impedimetric immunosensors	1–1000 ng·mL^−1^	1.0 ng·mL^−1^	-	-	Blood	[44]
Electrochemiscal immunobiosensor	0.01–10 ng·mL^−1^	10 pg·mL^−1^	10–15 min	-	Milk, stream water	[45]
Portable SPR sensor	26.4 ± 7.2 μg·L^−1^	2.0 ± 0.2 μg·L^−1^	<30 min	Once	Milk	[46]
SPR immunosensor	3.8 ng·mL^−1^	1.2 ng·mL^−1^	About 6 min	At least 100 times	Pure milk, egg, chicken muscle, beef and fish	This research

## Supplementary Materials

The supplementary materials are available online at http://www.mdpi.com/1424-8220/17/9/1984/s1. ESM. 1: UV adsorption spectra of ENRO, OVA and ENRO-OVA conjugates. ESM. 2: Inhibition curves of NOR, CIPRO, SPA and GAT using the developed SPR immunosensor.
