# Supplementary figures and images for: Development and Validation of a Reproducible and Label-Free Surface Plasmon Resonance Immunosensor for Enrofloxacin Detection in Animal-Derived Foods
Source: Sensors (Basel). 2017 Aug 30;17(9):1984. doi: 10.3390/s17091984 (PMC5621032; doi:10.3390/s17091984)

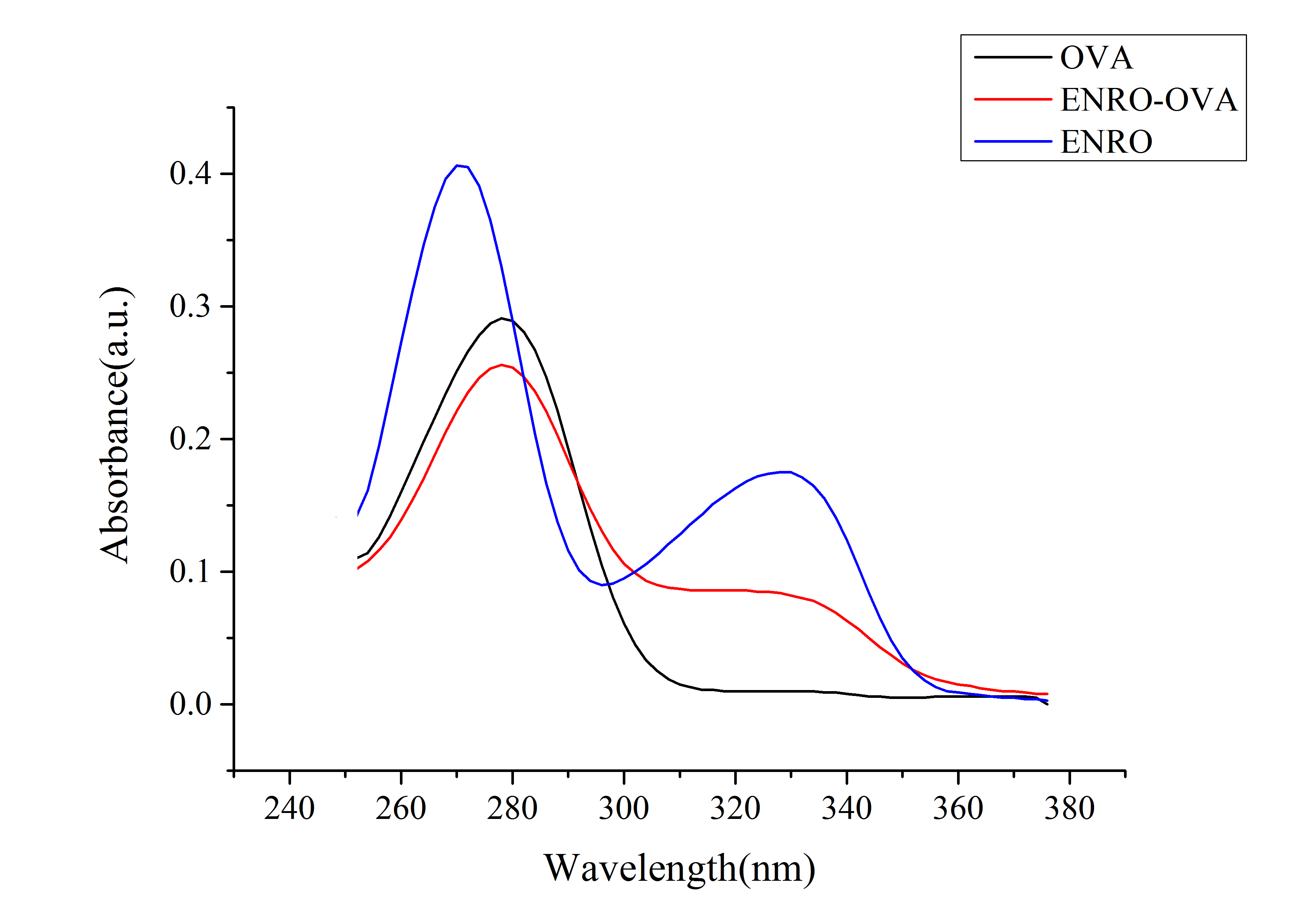

Supplement: Supplementary file 1 [file sensors-17-01984-s001.zip › ESM. 1.tif]
